# An extrauterine extensively metastatic epithelioid trophoblastic tumor responsive to pembrolizumab

**DOI:** 10.1016/j.gore.2021.100819

**Published:** 2021-06-23

**Authors:** Sarah G. Bell, Shitanshu Uppal, Michelle D. Sakala, Andrew P. Sciallis, Aimee Rolston

**Affiliations:** a University of Michigan Department of Obstetrics and Gynecology, 1500 E. Medical Center Dr., Ann Arbor, MI 48109, USA; b University of Michigan Department of Radiology, 1500 E. Medical Center Dr., Ann Arbor, MI 48109, USA; c University of Michigan Department of Pathology, 1500 E. Medical Center Dr., Ann Arbor, MI 48109, USA

**Keywords:** Epithelioid trophoblastic tumor, Gestational trophoblastic disease, Pembrolizumab, PD-L1 positivity

## Abstract

We report a case of extrauterine epithelioid trophoblastic tumor (ETT)—the rarest variant of gestational trophoblastic tumor—that has been stable on nearly two years of pembrolizumab treatment. A 47-year-old gravida 2, para 2 who underwent a prophylactic bilateral salpingo-oophorectomy nine years prior and bilateral mastectomy five years prior in the setting of a strong family history of breast and ovarian cancer with no genetic testing performed, presented to an outside clinic with recurrent respiratory infections without resolution despite antibiotics. Radiology and pathology testing confirmed the ETT diagnosis, including a second opinion from the John I. Brewer Trophoblastic Disease Center of Northwestern University’s Feinberg School of Medicine, and the patient was started on a chemotherapy regimen of etoposide, methotrexate, actinomycin-D, etoposide, and cisplatin for seven cycles, with partial improvement in her disease. After PD-L1 testing showed the tumor had > 5% PD-L1 positivity, she initiated pembrolizumab in April 2019. CT imaging after three months revealed decreased lung, abdominal, and pelvic disease and she was continued on pembrolizumab. As of December 2020, she had completed 29 cycles of pembrolizumab, with a plan for her to continue treatment indefinitely given her decreased, but persistent, disease. Our findings suggest pembrolizumab is a reasonable option for treatment of patients with significant PD-L1 positivity on testing of the tumor.

## Highlights

• We report on a case of epithelioid trophoblastic tumor that responded to pembrolizumab.
• Epithelioid trophoblastic tumor is the rarest variant of gestational trophoblastic disease.
• Immune checkpoint inhibitors are gaining popularity in patients with gestational trophoblastic disease.

## 1 Introduction

Epithelioid trophoblastic tumor (ETT) is the rarest variant of gestational trophoblastic disease, with tumor arising from intermediate trophoblastic cells of chorionic leave (Hui, 2019, Shih and Kurman, 1998). It was first described as a distinct entity in 1998, with approximately 130 total cases ever reported in the first 20 years since its identification (Shih and Kurman, 1998, Yang et al., 2019). ETTs present in women up to age 18 years following a gestation—typically after a full-term pregnancy, but also after a spontaneous abortion or hydatidiform mole. The majority of ETT cases present with irregular vaginal bleeding, but there have also been several documented extrauterine cases (Ahn et al., 2013, Davis et al., 2015, Kim et al., 2017, Kim et al., 2013, Shih and Kurman, 1998).

Due to limited cases and data, treatment for ETT has traditionally been similar to that for placental site trophoblastic disease. Among patients without metastatic disease, surgery is the primary treatment. Chemotherapy is used in patients with metastatic disease and/or in those who failed surgical intervention. Conventional chemotherapy has mixed success in treating patients with metastatic ETT; therefore, antiangiogenic and immunotherapy targeting immune checkpoints are being explored as alternative methods of treatment. The optimal chemotherapy regimen has not been identified, but 5-fluorouracil, actinomycin-D, etoposide, and vincristine (FAEV); etoposide, methotrexate, actinomycin-D, cyclophosphamide, and vincristine (EMA/CO); and etoposide, methotrexate, actinomycin-D, etoposide, and cisplatin (EMA/EP) have all been used, with rates of survival ranging from 64.5% to 72% depending on stage of disease, chemotherapeutic regimen, and time since follow-up (Frijstein et al., 2019, Yang et al., 2019).

As immune checkpoint inhibitors gain popularity, there is increasing interest in pathologic markers in gestational trophoblastic disease. A recent study looked at programmed cell death protein 1 (PD-L1) expression in gestational trophoblastic diseases and found that PD-L1 was highly expressed in syncytiotrophoblasts in complete molar pregnancies and choriocarcinomas. PD-L1 had variable expression in intermediate trophoblastic cells in chorionic laeve and implantation site (Veras et al., 2017). Another study of 21 patients with ETT reviewed pathologic markers and found several potential therapeutic targets including PD-L1, PD-L2, B7-H3, VISTA, and CD105 (Yang et al., 2019).

Pembrolizumab is an immune checkpoint inhibitor that targets PD-L1 to activate T-cell-mediated immune response against tumor cells. Pembrolizumab has been FDA-approved for use as targeted therapy in patients with recurrent or metastatic cervical cancer and certain endometrial tumors and is currently under trial for use in recurrent or progressive ovarian cancer (Grywalska et al., 2019, Makker et al., 2020, Marabelle et al., 2020). Since many gestational trophoblastic tumors have been shown to have strong PD-L1 expression, pembrolizumab has been hypothesized to be a treatment option for drug-resistant tumors (Bolze et al., 2017, Ghorani et al., 2017).

## 2 Case report

**Fig. 1:**
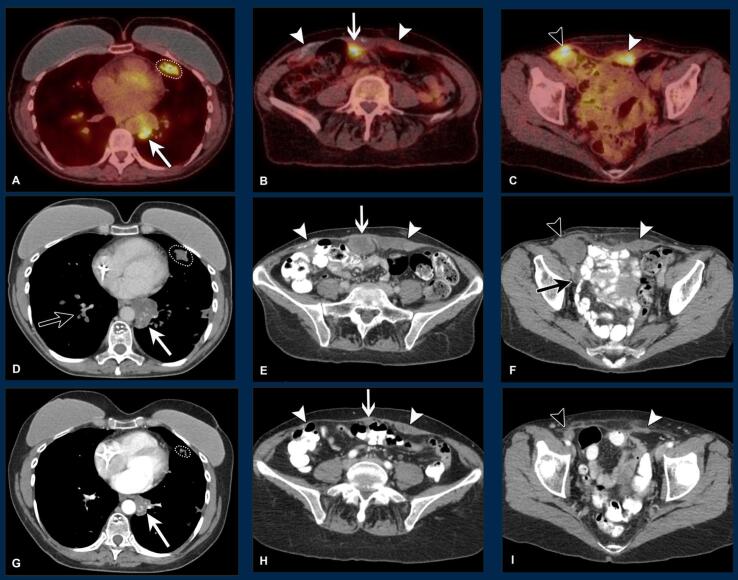
**Radiology images showing response over time in the chest, abdomen, and pelvis** A-C: Pretreatment 18F-FDG PET/CT imaging at initial workup: Metastatic disease seen as avid pulmonary nodules (A, circle); partially necrotic infrahilar mass (A, white closed arrow); peritoneal mass along abdominal wall (B, white open arrow); external iliac node (C, black arrowhead); and patchy areas of avidity within confluent thickening of the rectus abdominis muscles, where there are also dystrophic calcifications (B and C, white arrowheads). D-F: Axial post-contrast CT imaging eight months later: Progression after EMA/EP seen as continued pulmonary nodules (D, circle); enlarging infrahilar mass (D, white closed arrow; 4 cm, previously 3.4 cm); peritoneal mass (E, white open arrow; 4.1 cm, previously 2.8 cm); external iliac node (F, black arrowhead; measuring 3.9 cm, previously 2.7 cm); and continued rectus abdominis thickening (E and F, white arrowheads). Note also tumoral and bland pulmonary emboli (D, black closed arrow) and deep venous thrombosis (F, black closed arrow) from invasion of the right external iliac vein at the level of the external iliac node. G-I: Axial post-contrast CT imaging an additional five months later: Response with pembrolizumab with decreased pulmonary nodules (G, circle); infrahilar mass (G, white closed arrow; 2.3 cm, previously 4 cm); peritoneal mass (H, white open arrow; 1 cm, previously 3.9 cm); right inguinal node (I, black arrowhead; measuring 1.5 cm, previously 3.9 cm); and rectus abdominis thickening (H and I, white arrowheads).

**Fig. 2:**
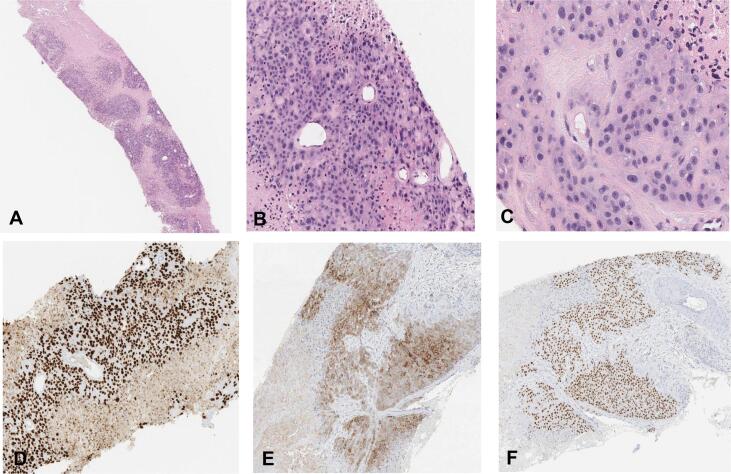
**Epithelioid trophoblastic tumor pathology slides** A: Core biopsy of ETT showing a nodular proliferation of plump epithelioid cells with eosinophilic cytoplasm separated by zones of necrosis (hematoxylin and eosin (H&E), 40X). B: This image highlights the nodular architecture of ETT. The nodules are often separated by zones of necrosis. At the center of each nodule is a small capillary (H&E, 200X). C: ETT often shows some variability in nuclear size and shape; however, mitotic figures are often inconspicuous. Of note, glandular differentiation and syncytiotrophoblastic cells are not identified (H&E, 400X). D: Like other types of trophoblastic lesions, ETT is often positive for GATA3. This marker is not specific for ETT, as expression can be seen in a wide variety of neoplasms including urothelial and mammary carcinomas (100X). E: The trophoblastic cells are positive for inhibin (100X). F: The trophoblastic cells are also positive for p63. ETT is thought to be derived from chorionic-type intermediate trophoblastic cells, which are usually positive for p63 (H&E, 100X). This is different from trophoblastic lesions derived from implantation-type intermediate trophoblastic cells, a group that includes placental site trophoblastic tumors (PSTT), which are usually negative for p63.

A 47-year-old gravida 2, para 2 who underwent a prophylactic bilateral salpingo-oophorectomy nine years prior and bilateral mastectomy five years prior in the setting of a strong family history of breast and ovarian cancer with no genetic testing performed, presented to an outside clinic with recurrent respiratory infections without resolution despite treatment with antibiotics. A chest CT in June 2018 showed bland and probable tumoral pulmonary emboli, pulmonary nodules and infarcts (some with cavitation or ground-glass halo), an infrahilar mass, and borderline enlarged lymph nodes. Fluorine-18 flurorodeoxyglucose PET/CT (^18^F-FDG PET/CT) in August 2018 revealed avidity in some pulmonary nodules, the infrahilar mass, areas of thickening and calcification of the rectus abdominis muscles, and a right external iliac lymph node with adjacent focal uptake within the external iliac vein (Fig. 1, A-C). There was no uptake in the uterus, cervix, or vagina or evidence of solid organ metastasis. Pathology from core needle biopsy of the external iliac lymph node was consistent with epithelioid trophoblastic tumor (Fig. 2, A-F). Beta hCG at that time was 9. Her last pregnancy was in 2006. A second opinion from the John I. Brewer Trophoblastic Disease Center of Northwestern University’s Feinberg School of Medicine was in agreement with the pathology diagnosis. The patient was started on EMA/EP and completed seven cycles, with partial improvement in her disease noted on follow-up ^18^F-FDG PET/CT scan in January 2019. She was not a surgical candidate due to extensive metastases in her thorax, intracardiac, and abdominal soft tissues.

She underwent PD-L1 testing that found the tumor had > 5% PD-L1 positivity using the Combined Positive Score. She initiated pembrolizumab in April 2019. CT imaging after three months on pembrolizumab revealed decreased lung, abdominal, and pelvic disease (Fig. 1, D-F). She was continued on pembrolizumab, with improvement of lung and abdominal disease on her next CT scan in July 2019 (Fig. 1, G-I). As of December 2020, she had completed 29 cycles of pembrolizumab, with beta hCG less < 2 since August 2019. Given her decreased but persistent disease while on pembrolizumab, the most recent Tumor Board consensus was to continue treatment with pembrolizumab.

## 3 Discussion

ETTs are extremely rare, with only two other case reports of ETT arising in post-menopausal women (Coulson et al., 2000, Park and Bae, 2016). The interval between prior gestation and diagnosis for our patient was 12 years, which is consistent with literature reporting a range of 1–18 years (Shih and Kurman, 1998). Typically, patients present with abnormal uterine bleeding, but this patient presented with recurrent respiratory infections and pulmonary embolism with infarcts in the setting of an extrauterine ETT. Our patient underwent a standard first line chemotherapy regimen of EMA/EP with minimal response.

Given that her tumor testing showed high PD-L1 positivity, she was started on pembrolizumab. After 28 cycles of pembrolizumab, her most recent imaging was consistent with decreased but persistent disease. Given these findings, our Tumor Board recommended continuation of treatment with pembrolizumab beyond the traditional 24 cycles. Although there is little known about the long-term impact of anti-PD-L1 therapy, a recent article published in *JAMA Oncology* found that, among patients on long term anti-PD-L1 therapy, 19.5% had a grade 3, 4, or 5 event and 43% had a chronic immune-related adverse event that continued 12 weeks beyond discontinuation of therapy (Patrinely et al., 2021).

Recently there has been a strong interest in using immunotherapy in patients with trophoblastic disease since a significant portion of trophoblastic cells express PD-L1 receptors (Veras et al., 2017). There have been reports of pembrolizumab successfully treating various forms of gestational trophoblastic diseases, including choriocarcinoma (Clair et al., 2020, Goldfarb et al., 2020). Additionally, there is a phase 2 clinical trial currently recruiting patients with chemo-resistant gestational trophoblastic disease for pembrolizumab treatment (https://clinicaltrials.gov/ct2/show/NCT04303884). There has been one published case report of a patient with metastatic ETT having significant decrease in disease while taking pembrolizumab (Huang et al., 2017). Another case series of four patients highlighted decreased disease burden for three patients on pembrolizumab (two with metastatic choriocarcinoma and one with metastatic placental site trophoblastic tumor). The fourth patient had a mixed placental site trophoblastic and epithelioid trophoblastic tumor; she had disease progression noted on pembrolizumab and succumbed to her disease (Ghorani et al., 2017). To the best of our knowledge, this is only the third report of a patient with metastatic ETT who was treated with pembrolizumab and only the second patient with resolution of extensive metastatic disease.

Based on our literature review using PubMed and Google Scholar, this case report is only the second to identify a patient with ETT who has responded to treatment with pembrolizumab. Our findings suggest pembrolizumab is a reasonable option for treatment of ETT in patients with significant PD-L1 positivity on testing of the tumor. Given the recent FDA approval for combination therapy with pembrolizumab, we anticipate continued advances in the treatment of ETT and other gestational trophoblastic diseases.

**Ethical Approval and Patient** Consent

The institutional review board at University of Michigan (HUM00193069, not regulated) approved this case report. The patient gave written and verbal consent for her clinical details and photos to be shared.

## CRediT authorship contribution statement

**Sarah G. Bell:** Conceptualization, Data curation, Investigation, Project administration, Resources, Validation, Writing - original draft, Writing - review & editing. **Shitanshu Uppal:** Conceptualization, Supervision, Writing - review & editing. **Michelle D. Sakala:** Data curation, Resources, Visualization, Writing - review & editing. **Andrew P. Sciallis:** Data curation, Resources, Visualization, Writing - review & editing. **Aimee Rolston:** Conceptualization, Investigation, Supervision, Validation, Writing - original draft, Writing - review & editing.

## Declaration of Competing Interest

The authors declare that they have no known competing financial interests or personal relationships that could have appeared to influence the work reported in this paper.
